# Efficacy of alirocumab according to background statin type and dose: pooled analysis of 8 ODYSSEY Phase 3 clinical trials

**DOI:** 10.1038/srep45788

**Published:** 2017-04-04

**Authors:** Alberico L. Catapano, L. Veronica Lee, Michael J. Louie, Desmond Thompson, Jean Bergeron, Michel Krempf

**Affiliations:** 1 Department of Pharmacological and Biomolecular Sciences, Università di Milano and IRCCS Multimedica, Milan, Italy; 2 Sanofi, Bridgewater, New Jersey, USA; 3 Regeneron Pharmaceuticals, Inc., Tarrytown, New York, USA; 4 Clinique des Maladies Lipidiques, CHU de Québec-Université Laval, Québec, QC, Canada; 5 CHU de Nantes – Hôpital Nord Laennec, Saint-Herblain, France

## Abstract

Low-density lipoprotein cholesterol (LDL-C) reductions with the PCSK9 monoclonal antibody alirocumab may be affected by background statin dose due to increased PCSK9 levels with higher statin doses. Data from 8 Phase 3 trials conducted with background statin (n = 4629) were pooled by alirocumab dose (75 or 150 mg every 2 weeks) and control (placebo/ezetimibe), and analyzed by background statin type/dose. Overall, 58.4% received high-dose statins (atorvastatin 40–80 mg, rosuvastatin 20–40 mg, simvastatin 80 mg), 28.6% moderate-dose statins (atorvastatin 20–<40 mg, rosuvastatin 10–<20 mg, simvastatin 40–<80 mg), and 12.9% low-dose statins (atorvastatin <20 mg, rosuvastatin <10 mg, simvastatin <40 mg). Mean baseline PCSK9 levels were higher with high versus moderate and low statin doses (318.5 vs 280.6 ng/mL). Baseline LDL-C levels were similar across pools, regardless of statin intensity. No associations were observed between statin type/dose and LDL-C % change from baseline or % of patients achieving LDL-C goals at Week 24 for alirocumab versus control (interaction *P*-values non-significant). Incidence of adverse events was similar for alirocumab versus control, except for a higher rate of injection-site reactions with alirocumab. In summary, alirocumab provided consistent LDL-C reductions and was generally well tolerated independent of background statin type/dose.

Statins (3-hydroxy-3-methyl-glutaryl-coenzyme A reductase inhibitors) are currently the first-line therapy for reducing levels of low-density lipoprotein cholesterol (LDL-C) and thus reducing cardiovascular risk^1^,^2^,^3^. However, not all patients achieve sufficient LDL-C-lowering on statin therapy alone, such as those with atherosclerotic cardiovascular disease (ASCVD), high baseline LDL-C levels (particularly patients with heterozygous familial hypercholesterolaemia [HeFH])^4^, or patients unable to tolerate high statin doses^5^,^6^,^7^. For patients who require LDL-C reduction beyond that achieved with statin therapy, recent updates to lipid management guidelines in the USA and Europe have proposed that adding non-statin therapies such as ezetimibe (a cholesterol absorption inhibitor) or an inhibitor of a proprotein convertase subtilisin/kexin type 9 (PCSK9) may be considered, depending on the patient’s risk level^1^,^8^.

PCSK9 binds to and promotes the degradation of LDL receptors on hepatocytes, resulting in fewer receptors being available to remove LDL-C from the circulation^9^. Inhibition of PCSK9 with the monoclonal antibody alirocumab reduces LDL-C levels by ~50–60% when added to a statin (with or without other lipid-lowering therapy [LLT]) in Phase 3 clinical trials^10^,^11^,^12^,^13^,^14^,^15^. The safety profile of alirocumab in those trials was generally comparable with placebo or ezetimibe controls, except for an increased incidence of injection-site reactions observed in alirocumab-treated patients. Alirocumab was approved in the USA and Europe in 2015 for treating high-risk patients who require additional reduction in LDL-C beyond that achieved with maximally tolerated statin and other LLTs^16^,^17^.

Clearance of alirocumab from the circulation is thought to be partly related to the concentration of PCSK9, through a phenomenon known as target-mediated clearance^18^. Therefore, higher PCSK9 concentrations through increased PCSK9 production are thought to increase the clearance of alirocumab^19^. Statin therapy increases circulating levels of PCSK9 through the statin-mediated activation of the transcription factor sterol regulatory element-binding protein-2, which leads not only to increased expression of the *LDLR* gene but also of the *PCSK9* gene^9^,^20^. The efficacy of monoclonal antibodies to PCSK9 could therefore potentially be impacted by higher versus lower statin doses due to increased PCSK9 levels and target-mediated clearance^19^. We investigated whether LDL-C reductions following alirocumab treatment were affected by background statin dose and type of statin, using pooled data from the ODYSSEY clinical trials programme which was mainly conducted on a background of maximally tolerated statin.

## Methods

### Study design and pooling strategy

**Figure 1:**
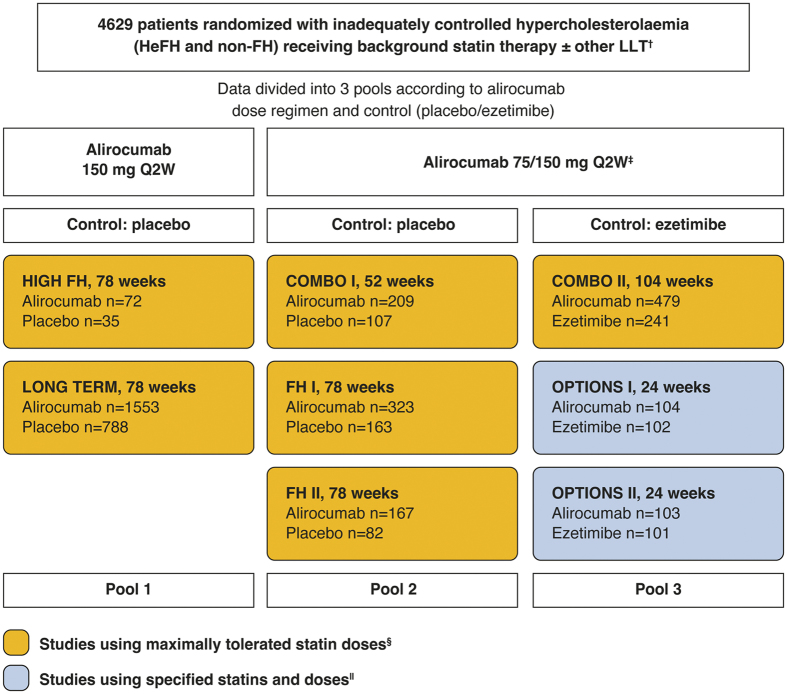
Overview of the Phase 3 ODYSSEY trials included in the analysis and pooling strategy. The number of patients randomized are indicated by n values. For purposes of this analysis, efficacy data were analyzed in 3 pools according to alirocumab dose (75/150 mg or 150 mg Q2W) and control (ezetimibe or placebo). For safety analysis, placebo-controlled studies (Pool 1 and Pool 2) were combined. ^†^Other LLTs not allowed at study entry in COMBO II. ^‡^The alirocumab dose was increased from 75 to 150 mg Q2W at Week 12 if LDL-C was ≥70 mg/dL at Week 8 (or ≥70 or ≥100 mg/dL in the OPTIONS studies depending on cardiovascular risk). ^§^Maximally tolerated statin was defined as atorvastatin 40–80 mg, rosuvastatin 20–40 mg, or simvastatin 80 mg, or lower doses with an investigator-approved reason. ^||^Atorvastatin 20–40 mg in OPTIONS I and rosuvastatin 10–20 mg in OPTIONS II. HeFH, heterozygous familial hypercholesterolaemia; LDL-C, low-density lipoprotein cholesterol; LLT, lipid-lowering therapy; Q2W, every 2 weeks. Clinicaltrials.gov identifiers: HIGH FH, NCT01617655^15^; LONG TERM, NCT01507831^14^; COMBO I, NCT01644175^11^; FH I, NCT01623115^13^; FH II, NCT01709500^13^; COMBO II, NCT01644188^11^; OPTIONS I, NCT01730040^10^; OPTIONS II, NCT01730053^12^.

This analysis includes data from 8 Phase 3 randomized, multicentre, double-blind, controlled trials which utilized background statin therapy (Fig. 1). Trial methods and primary results have been reported previously^10^,^11^,^12^,^13^,^14^,^15^. The trials were conducted in accordance with the Declaration of Helsinki and applicable amendments and International Conference Harmonization guidelines for Good Clinical Practice. Trial protocols were approved by the appropriate institutional review board or independent ethics committee, and written informed consent was obtained from all patients. All trials recruited patients at high ASCVD risk, with 3 trials (FH I, FH II, and HIGH FH) exclusively recruiting patients with HeFH^13^,^15^.

**Figure 2:**
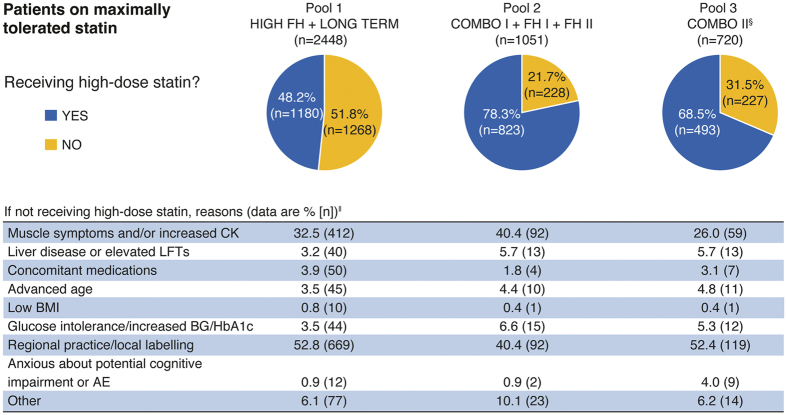
Investigator-approved reasons why patients were not receiving a high-dose statin^†^ in studies requiring participants to be on maximally tolerated statin^‡^. ^†^High dose statin defined as: atorvastatin 40–80 mg, rosuvastatin 20–40 mg, or simvastatin 80 mg. ^‡^All patients in Pool 1 and 2 and patients from COMBO II in Pool 3 were required to be on maximally tolerated statin at study entry, ideally a high-dose statin although lower doses were allowed with an investigator-approved reason. ^§^OPTIONS I and II not included as patients received study-defined doses of background statin rather than maximally tolerated doses. ^||^A patient can be counted in several categories. AE, adverse event; BG, blood glucose; BMI, body mass index; CK, creatine kinase; HbA1c, glycated haemoglobin; LFT, liver function test.

Inclusion criteria for 6 of the trials (LONG TERM, HIGH FH, FH I, FH II, COMBO I, and COMBO II) stipulated that patients were on maximally tolerated statin therapy. To meet the maximally tolerated statin criterion, patients were to be receiving the highest available statin doses (atorvastatin 40–80 mg, rosuvastatin 20–40 mg, or simvastatin 80 mg). Lower doses were allowed if an investigator-approved reason was given, such as statin intolerance or regional practice (see list in Fig. 2). Lower doses included moderate and low statin doses as well as off-label doses such as 5 mg/week (refer to Table 1 for moderate and low-dose statin definitions). In the other 2 trials, patients received pre-specified background statin therapy: atorvastatin 20 or 40 mg in OPTIONS I and rosuvastatin 10 or 20 mg in OPTIONS II.

To be eligible for each study, LDL-C levels had to be ≥70 mg/dL for patients with prior cardiovascular events and ≥100 mg/dL for those with no prior ASCVD but with other risk factors, with the exception of LONG TERM and HIGH FH, in which LDL-C criteria were ≥70 and ≥160 mg/dL for all patients, respectively. Patients with triglycerides >400 mg/dL were excluded from all studies.

Patients were randomized to either alirocumab or control (placebo or ezetimibe) in a 2:1 ratio (except for OPTIONS I and II where a 1:1 ratio was used). The double-blind treatment period lasted 24–104 weeks. For the current analyses, efficacy data were grouped into three pools according to initial alirocumab dose and control (Fig. 1). Two trials compared alirocumab 150 mg administered every 2 weeks (Q2W) versus placebo (Pool 1). The other 2 pools used a dose increase strategy (indicated in the text by 75/150 mg Q2W) whereby the initial dose of alirocumab 75 mg was increased to 150 mg at Week 12 if Week 8 LDL-C levels exceeded protocol-defined thresholds (≥70 mg/dL in all studies except OPTIONS I and II, where the threshold was LDL-C ≥ 70 mg/dL or ≥100 mg/dL based on patients’ risk level)^10^,^12^. Three trials compared alirocumab 75/150 mg Q2W versus placebo (Pool 2), and 3 trials compared alirocumab 75/150 mg Q2W versus ezetimibe (Pool 3). Baseline and efficacy data were analyzed in subgroups according to background statin type and dose. Safety data were pooled and analyzed according to whether the study was placebo- or ezetimibe-controlled and according to background statin dose.

### LDL-C and PCSK9 analysis

Lipid analyses were performed using standardized methods by a central laboratory (Medpace Reference Laboratories in all studies, except for LONG TERM, which used Covance Central Laboratories). Total cholesterol, triglycerides, and high-density lipoprotein cholesterol (HDL-C) levels in serum were determined using Centers for Disease Control and Prevention National Heart Lung Blood Institute Lipid Standardization Program assays. LDL-C was calculated using the Friedewald formula (total cholesterol − HDL-C − triglycerides/5). LDL-C was also measured via ultracentrifugation and precipitation (beta-quantification) by the central laboratory in cases where triglyceride values were >400 mg/dL. Free PCSK9 concentrations in serum (unbound to alirocumab or LDL receptors) were determined using a validated enzyme-linked immunosorbent assay method (FH I, COMBO II, and LONG TERM studies only; Regeneron Pharmaceuticals Inc., Tarrytown, NY, USA).

### Endpoints and statistical analysis

Efficacy endpoints included the mean percent age change in LDL-C from baseline to Week 24 (this was also the primary endpoint in each study), and the proportion of patients achieving risk-based LDL-C goals. Data were analyzed using an intent-to-treat approach, including all lipid data regardless of treatment adherence. LDL-C percent age change was assessed using a mixed-effect model with repeated measures (MMRM) analysis to account for missing values. The impact of background statin dose was assessed in individual pools by comparing the difference (alirocumab vs control) in LDL-C percent age change between subgroups of patients according to the statin type and dose received, using the same MMRM analysis as above. The proportions of patients achieving LDL-C goals were estimated from multiple imputation. To assess the impact of baseline parameters (distance to LDL-C goal, PCSK9, and statin dose) on LDL-C goal achievement, odds ratios and P-values were calculated using multivariate logistic regression. Safety was assessed via reporting of treatment-emergent adverse events (TEAEs) and laboratory values. Adverse events were classed as TEAEs if they were reported from the first dose of study treatment up to the last dose plus 70 days. Only descriptive statistics were used for the safety analyses (no formal statistics were planned in the study protocols).

## Results

This analysis included 4629 patients who were randomized in 8 trials (Fig. 1).

### Patient baseline characteristics

Demographic and baseline characteristics were well-balanced between the alirocumab and control groups within each pool (Table 1). Differences in baseline characteristics between pools reflected the inclusion criteria of the studies comprising each pool. Pool 2 (which included 2 studies performed exclusively in HeFH patients) had the lowest proportion of ASCVD and diabetes mellitus and the highest proportion of HeFH (Table 1). Patients in Pool 2 also had a lower mean age, a higher mean baseline LDL-C, and a greater proportion of patients on the highest doses of statins compared with the other pools (Table 1).

Overall, 2704/4629 patients (58.4%) were receiving high-dose statins (atorvastatin 40–80 mg, rosuvastatin 20–40 mg, simvastatin 80 mg), 28.6% were receiving moderate-dose statins (atorvastatin 20–<40 mg, rosuvastatin 10–<20 mg, or simvastatin 40–<80 mg), and 12.9% were receiving low-dose statins (atorvastatin <20 mg, rosuvastatin <10 mg, or simvastatin <40 mg). Differences in baseline characteristics between patients receiving high-dose statins compared with those not on high doses of statins are shown in Table 2. Notably, the proportion of HeFH was higher and diabetes mellitus was lower in the high-dose statin pool; also, baseline LDL-C and PCSK9 values were higher in the high-dose statin pool (Table 2). The proportion of patients on non-statin LLTs was also higher in the high-dose statin pool (Table 2).

### Utilization of maximally tolerated statin therapy

All patients in the studies in Pools 1 and 2 as well as 69.8% of patients in Pool 3 (all from the COMBO II study) were on maximally tolerated statin, as required by study inclusion criteria (Table 1). The percentage of these patients receiving the highest doses of statins ranged from 48.2% to 78.3% across the pooled groups (Fig. 2). The most common reasons for not receiving the highest doses were regional practice or local labelling, and history of muscle symptoms and/or increase in creatine kinase levels (Fig. 2). Among patients receiving moderate or low doses of statins, a small proportion (34/4629, 0.7%) were receiving non-standard or off-label doses of statins (Supplementary Table 1).

### LDL-C reductions

**Figure 3:**
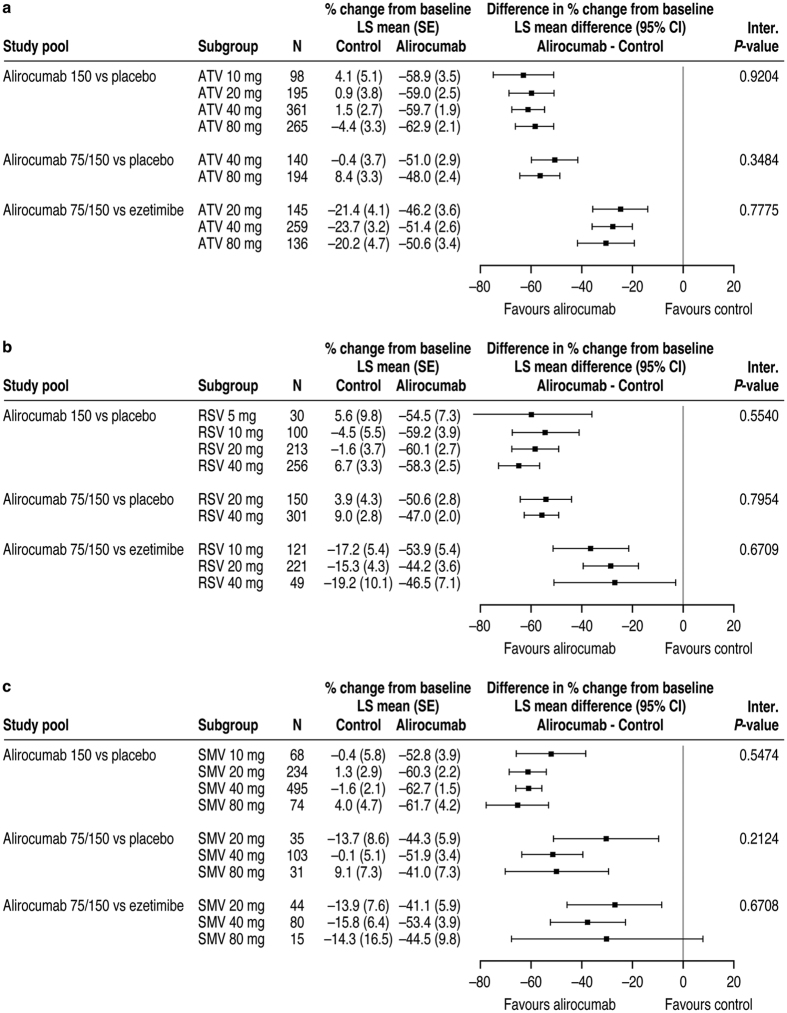
Percent age change from baseline in LDL-C at Week 24: Subgroup analysis by (**a**) atorvastatin dose, (**b**) rosuvastatin dose, and (**c**) simvastatin dose (ITT analysis). For each statin type, alirocumab data were analyzed in 3 pools according to alirocumab dose and control. In panel c, simvastatin data for the ALI 75/150 mg Q2W versus ezetimibe study pool are for the COMBO II trial only (simvastatin not used in the OPTIONS studies). ATV, atorvastatin; CI, confidence interval; ITT, intent-to-treat; LS, least squares; RSV, rosuvastatin; SE, standard error; SMV, simvastatin.

Alirocumab dose was increased from 75 to 150 mg Q2W at Week 12 in 35% and 18% of patients in Pools 2 and 3, respectively. Alirocumab treatment produced greater LDL-C reductions compared with controls across all types and doses of background statins (Fig. 3). Across all study pools, no association was observed between statin dose and the difference in LDL-C percent age change for alirocumab versus control at Week 24 (interaction P-values not significant; Fig. 3). Absolute reductions in LDL-C also did not appear to be affected by statin type and dose (Supplementary Table 2).

### LDL-C goal attainment

**Figure 4:**
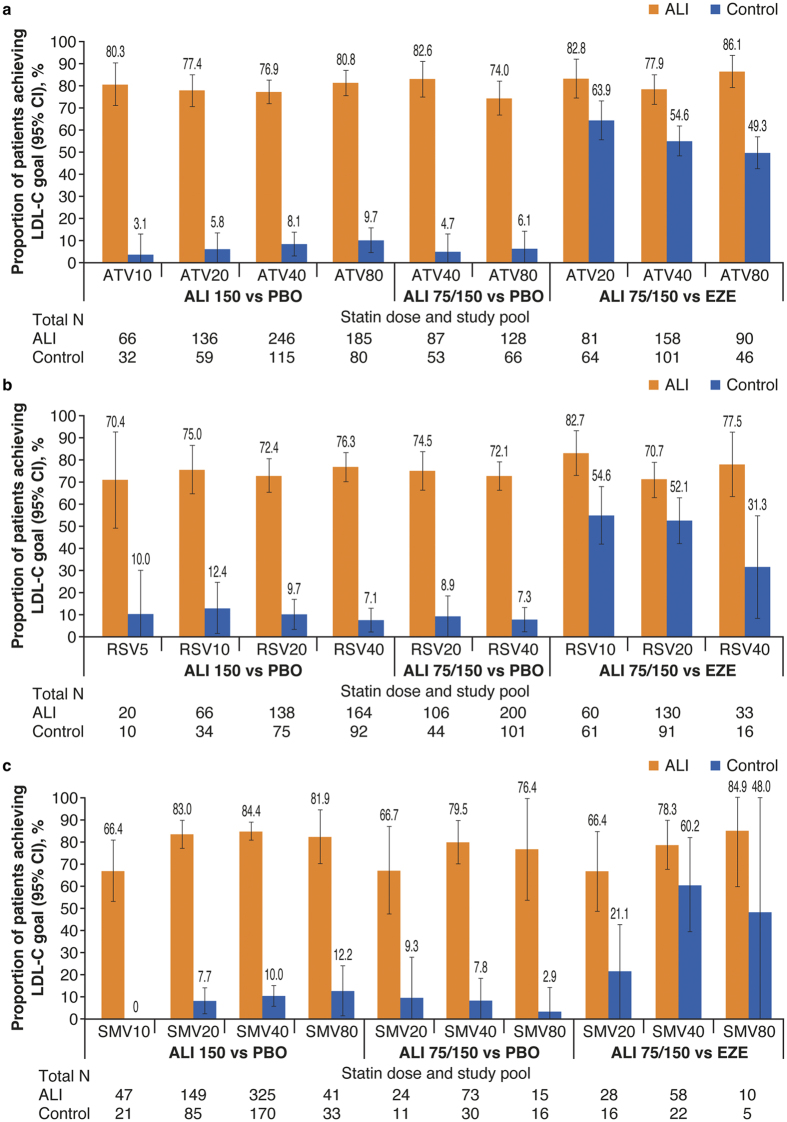
Proportion of patients achieving LDL-C goals of <70 or <100 mg/dL (goal determined by cardiovascular risk): Subgroup analysis by statin and (**a**) atorvastatin dose, (**b**) rosuvastatin dose, and (**c**) simvastatin dose (ITT analysis). For each statin type, efficacy data were analyzed in 3 pools according to ALI dose and control. In panel c, simvastatin data for the alirocumab 75/150 mg Q2W versus ezetimibe study pool are for the COMBO II trial only (simvastatin not used in the OPTIONS studies). All interaction P-values comparing doses of each statin within each pool were not significant. ALI, alirocumab; ATV, atorvastatin; CI, confidence interval; EZE, ezetimibe; ITT, intent-to-treat; LDL-C, low-density lipoprotein cholesterol; PBO, placebo; Q2W, every 2 weeks; RSV, rosuvastatin; SMV, simvastatin.

The proportion of patients achieving LDL-C goals (depending on cardiovascular risk) was consistently higher with alirocumab versus control for each statin dose, with no relationship between statin dose and goal achievement (Fig. 4 and Table 3). Patients were less likely to attain their LDL-C goal on alirocumab the further away their baseline LDL-C was from risk-based goal (Table 3). There was a pattern for higher baseline PCSK9 levels being associated with increased likelihood of goal attainment; however, this was not significant (Table 3).

### Safety summary

The incidence of TEAEs, serious adverse events, and TEAEs leading to death or discontinuation was similar between patients who received alirocumab and control, except for a higher rate of injection-site reactions with alirocumab (Table 4). There were no major differences in the TEAE profile between patients receiving higher and lower doses of statins (Table 4).

## Discussion

The current analysis tested the hypothesis that the LDL-C-lowering efficacy of a given dose of alirocumab may be reduced when administered with a higher versus lower dose of statin therapy, due to increased PCSK9 levels at higher statin doses. Patients in the trials included in this analysis who were receiving high-dose statins tended to have higher baseline LDL-C and PCSK9 levels compared with those not on high-dose statins, in agreement with previous reports^9^,^20^. Regardless, the differences in statin dose intensity did not appear to clinically impact the magnitude of LDL-C reductions observed following treatment with alirocumab 75 or 150 mg Q2W, nor was achievement of LDL-C goals affected by statin dose intensity. In support of these results, a previous small study suggested slightly higher alirocumab efficacy when dosed 150 mg Q2W with a statin compared with no statin (LDL-C reductions from baseline of 65.7% and 57.0%, respectively)^21^. Background statin seems to have more of an impact on alirocumab efficacy when longer dosing intervals are used (every 4 weeks [Q4W] vs Q2W). Phase 2 studies indicated that efficacy was not fully maintained over the dosing interval when alirocumab 150 mg Q4W was co-administered with a statin^21^,^22^, probably because of statin-induced increases in PCSK9 levels leading to increased alirocumab clearance^19^. However, efficacy was stable with the 150 mg Q2W dose when co-administered with a statin^21^,^22^, and has also been shown to be relatively stable with a dose of 300 mg Q4W^23^.

Differences in baseline PCSK9 levels did not have a significant effect on achievement of LDL-C goals in alirocumab-treated patients. The main driver behind achieving LDL-C goals with alirocumab was baseline LDL-C (and distance to LDL-C goal). Similar findings were reported previously in a pooled analysis of 6 alirocumab trials^24^.

Limitations of this analysis include the relatively low number of patients who received some statin doses or types, and that patients were not randomized to their background statin dose and type. There were differences between patients on high-dose statins versus lower doses that probably result from differences in patient populations and trial recruitment criteria, e.g. patients receiving the higher statin doses tended to have HeFH and were younger, with higher baseline LDL-C and a lower frequency of ASCVD and diabetes, compared with patients on lower statin doses. Hence the analysis is not only comparing just statin doses but also somewhat different patient populations. Also, assessment of the impact of PCSK9 levels on efficacy is limited as PCSK9 data were only available for 3 of the 8 studies analyzed here. This analysis evaluated alirocumab 75 or 150 mg when dosed Q2W; however, it is possible that statin type and dose may affect alirocumab efficacy if a longer dosing interval or lower dose were used.

Similar LDL-C reductions were also observed regardless of background statin type or dose following treatment with another PCSK9 inhibitor (evolocumab) in the LAPLACE-2 study^25^.

To conclude, regardless of the statin dose and type (atorvastatin, rosuvastatin, or simvastatin), the tested doses of alirocumab (75 or 150 mg Q2W) provided consistent reductions in LDL-C, and enabled a significantly greater proportion of patients to achieve their LDL-C goals than either placebo or ezetimibe control. Alirocumab was generally well tolerated compared with controls, regardless of background statin dose.

## Additional Information

**How to cite this article:** Catapano, A. L. *et al*. Efficacy of alirocumab according to background statin type and dose: pooled analysis of 8 ODYSSEY Phase 3 clinical trials. *Sci. Rep.* **7**, 45788; doi: 10.1038/srep45788 (2017).

**Publisher's note:** Springer Nature remains neutral with regard to jurisdictional claims in published maps and institutional affiliations.

## Supplementary Material

Supplementary Information

## Figures and Tables

**Table 1:** Baseline characteristics of randomized patients by analysis pool.

	Pool 1		Pool 2		Pool 3	
	Alirocumab 150 mg Q2W (n = 1625)	Placebo (n = 823)	Alirocumab 75/150 mg Q2W (n = 699)	Placebo (n = 352)	Alirocumab 75/150 mg Q2W (n = 686)	Ezetimibe (n = 444)
Age (years), mean ± SD	60.0 ± 10.8	60.2 ± 10.6	55.6 ± 12.9	55.5 ± 12.5	61.6 ± 9.7	62.3 ± 9.7
Male, n (%)	1018 (62.6)	496 (60.3)	397 (56.8)	216 (61.4)	483 (70.4)	294 (66.2)
Race (white), n (%)	1505 (92.6)	760 (92.3)	634 (90.7)	312 (88.6)	582 (84.8)	385 (86.7)
BMI (kg/m^2^), mean ± SD	30.1 ± 5.7	30.5 ± 5.4	30.0 ± 5.5	30.1 ± 6.0	30.3 ± 5.9	30.7 ± 5.6
HeFH, n (%)	348 (21.4)	174 (21.1)	490 (70.1)	245 (69.6)	26 (3.8)	18 (4.1)
Diabetes mellitus, n (%)	566 (34.8)	284 (34.5)	133 (19.0)	71 (20.2)	244 (35.6)	167 (37.6)
ASCVD, n (%)	1219 (75.0)	634 (77.0)	396 (56.7)	200 (56.8)	580 (84.5)	353 (79.5)
CHD, n (%)	1085 (66.8)	574 (69.7)	369 (52.8)	192 (54.5)	547 (79.7)	336 (75.7)
ACS, n (%)	734 (45.2)	394 (47.9)	246 (35.2)	134 (38.1)	402 (58.6)	241 (54.3)
Calculated LDL-C (mg/dL), mean ± SD	125.9 ± 45.9	125.3 ± 44.5	129.0 ± 47.3	130.3 ± 45.4	109.4 ± 35.6	105.0 ± 36.2
Background therapy, n (%)						
Use of any statin	1624 (>99.9)	822 (99.9)	698 (99.9)†	351 (99.7)	685 (99.9)	444 (100)
Maximally tolerated statin‡	1625 (100)	823 (100)	699 (100)	352 (100)	479 (69.8)	241 (54.3)
High-dose statin§	785 (48.3)	400 (48.7)	542 (77.8)	282 (80.3)	430 (62.8)	265 (59.7)
Moderate-dose statin||	542 (33.4)	271 (33.0)	108 (15.5)	43 (12.3)	208 (30.4)	152 (34.2)
Low-dose statin¶	297 (18.3)	151 (18.4)	47 (6.7)	26 (7.4)	47 (6.9)	27 (6.1)
Non-statin LLT	487 (30.0)	244 (29.6)	395 (56.5)	217 (61.6)	75 (10.9)	55 (12.4)

^†^One patient was receiving pravastatin and is not included in the counts for high/moderate/low-dose statins.

^‡^All patients in Pool 1 and 2 and patients from COMBO II in Pool 3 were required to be on maximally tolerated statin at study entry, ideally a high-dose statin although lower doses were allowed with an investigator-approved reason.

^§^High-dose statin: atorvastatin 40–80 mg, rosuvastatin 20–40 mg, or simvastatin 80 mg.

^||^Moderate-dose statin: atorvastatin 20–<40 mg, rosuvastatin 10–<20 mg, or simvastatin 40–<80 mg.

^¶^Low-dose statin: atorvastatin <20 mg, rosuvastatin <10 mg, or simvastatin <40 mg.

Studies included in each pool: 1: HIGH FH and LONG TERM; 2: COMBO I, FH I, and FH II; 3: COMBO II, OPTIONS I, and OPTIONS II. ACS, acute coronary syndrome; ASCVD, atherosclerotic cardiovascular disease (includes CHD, ischaemic stroke, transient ischaemic attack, and peripheral arterial disease); BMI, body mass index; CHD, coronary heart disease; HeFH, heterozygous familial hypercholesterolaemia; LDL-C, low-density lipoprotein cholesterol; LLT, lipid-lowering therapy; Q2W, every 2 weeks; SD, standard deviation.

**Table 2:** Baseline characteristics of randomized patients according to intensity of background statin dose.

	High-dose statin (n = 2704)	Not high-dose statin (n = 1925)	P-value
Age (years), mean ± SD	58.3 ± 11.2	61.1 ± 10.9	0.0001
Male, n (%)	1731 (64.0)	1173 (60.9)	0.0066
Race (white), n (%)	2493 (92.2)	1685 (87.5)	<0.0001
BMI (kg/m^2^), mean ± SD	30.0 ± 5.4	30.6 ± 6.0	0.0442
HeFH, n (%)	1023 (37.8)	278 (14.4)	<0.0001
Diabetes mellitus, n (%)	691 (25.6)	774 (40.2)	<0.0001
ASCVD, n (%)	2016 (74.6)	1366 (71.0)	<0.0001
CHD, n (%)	1883 (69.6)	1220 (63.4)	<0.0001
ACS, n (%)	1326 (49.0)	825 (42.9)	<0.0001
Baseline LDL-C (mg/dL), mean ± SD	125.2 ± 45.9	117.8 ± 41.7	0.0034
Baseline free PCSK9 levels (ng/mL), mean ± SD^†^	318.5 ± 126.8	280.6 ± 103.7	<0.0001
Non-statin LLT, n (%)	1063 (39.3)	410 (21.3)	<0.0001

^†^PCSK9 levels were available only for studies FH I, COMBO II, and LONG TERM.

Pool of FH I, FH II, COMBO I, COMBO II, LONG TERM, HIGH FH, OPTIONS I, and OPTIONS II. High dose statin defined as: atorvastatin 40–80 mg, rosuvastatin 20–40 mg, or simvastatin 80 mg. P-values for continuous variables based on analysis of variance, adjusted on study. P-values for categorical variables based on Cochran-Mantel-Haenszel test, stratified on study. ACS, acute coronary syndrome; ASCVD, atherosclerotic cardiovascular disease (includes CHD, ischaemic stroke, transient ischaemic attack, and peripheral arterial disease); BMI, body mass index; CHD, coronary heart disease; HeFH, heterozygous familial hypercholesterolaemia; LDL-C, low-density lipoprotein cholesterol; LLT, lipid-lowering therapy; PCSK9, proprotein convertase subtilisin/kexin type 9; SD, standard deviation.

**Table 3:** Predictive factors of achieving LDL-C goal at Week 24 – multivariate analysis in patients randomized to alirocumab.

Factor	Category	N	Number (%) of patients achieving LDL-C goal	Odds ratio (95% CI)	P-value
Distance to LDL-C goal†	<30 mg/dL (ref)	832	745 (89.5)	—	<0.0001
	≥30–<60 mg/dL	801	675 (84.3)	0.60 (0.44–0.81)	
	≥60–<90 mg/dL	370	251 (67.8)	0.25 (0.18–0.35)	
	≥90 mg/dL	316	164 (51.9)	0.13 (0.10–0.18)	
Baseline free PCSK9	<200 ng/mL (ref)	424	301 (71.0)	—	0.1449
	≥200–<300 ng/mL	815	655 (80.4)	1.26 (0.94–1.68)	
	≥300–<400 ng/mL	573	465 (81.2)	1.28 (0.93–1.76)	
	≥400 ng/mL	421	354 (84.1)	1.51 (1.05–2.17)	
Statin treatment	High-dose (ref)	1310	1022 (78.0)	—	0.2419
	Not high-dose	1009	813 (80.6)	1.14 (0.91–1.43)	

^†^Calculated as baseline LDL-C minus risk-based LDL-C goal.

Pool of FH I, COMBO II, and LONG TERM. Odds ratios and P-value calculated from a multivariate logistic regression. Patients with missing PCSK9 levels were excluded from the multivariate analysis. CI, confidence interval; ITT, intent-to-treat; LDL-C, low-density lipoprotein cholesterol; PCSK9, proprotein convertase subtilisin/kexin type 9.

**Table 4:** Safety summary.

Data are n (%)	Placebo-controlled pool n = 3492				Ezetimibe-controlled pool n = 1129			
Pooled treatment group	Alirocumab n = 2318		Placebo n = 1174		Alirocumab n = 686		Ezetimibe n = 443	
Statin dose subgroup	High-dose n = 1325	Not high-dose n = 993	High-dose n = 682	Not high-dose n = 492	High-dose n = 430	Not high-dose n = 256	High-dose n = 264	Not high-dose n = 179
TEAEs	1041 (78.6)	810 (81.6)	543 (79.6)	411 (83.5)	333 (77.4)	184 (71.9)	188 (71.2)	129 (72.1)
Treatment-emergent SAEs	219 (16.5)	166 (16.7)	119 (17.4)	83 (16.9)	90 (20.9)	44 (17.2)	43 (16.3)	32 (17.9)
TEAEs leading to death	7 (0.5)	9 (0.9)	7 (1.0)	6 (1.2)	3 (0.7)	3 (1.2)	5 (1.9)	4 (2.2)
TEAEs leading to discontinuation	66 (5.0)	78 (7.9)	42 (6.2)	25 (5.1)	31 (7.2)	25 (9.8)	15 (5.7)	16 (8.9)
TEAEs in ≥5% of patients								
Nasopharyngitis	164 (12.4)	127 (12.8)	69 (10.1)	73 (14.8)	24 (5.6)	8 (3.1)	13 (4.9)	10 (5.6)
Injection-site reaction	108 (8.2)	59 (5.9)	40 (5.9)	22 (4.5)	13 (3.0)	5 (2.0)	3 (1.1)	2 (1.1)
Influenza	86 (6.5)	61 (6.1)	34 (5.0)	29 (5.9)	20 (4.7)	9 (3.5)	10 (3.8)	8 (4.5)
Upper respiratory tract infection	81 (6.1)	81 (8.2)	56 (8.2)	38 (7.7)	34 (7.9)	19 (7.4)	16 (6.1)	14 (7.8)
Arthralgia	70 (5.3)	48 (4.8)	40 (5.9)	36 (7.3)	14 (3.3)	18 (7.0)	10 (3.8)	5 (2.8)
Back pain	66 (5.0)	57 (5.7)	32 (4.7)	38 (7.7)	18 (4.2)	9 (3.5)	5 (1.9)	11 (6.1)
Bronchitis	68 (5.1)	44 (4.4)	29 (4.3)	29 (5.9)	13 (3.0)	8 (3.1)	8 (3.0)	5 (2.8)
Urinary tract infection	68 (5.1)	60 (6.0)	27 (4.0)	38 (7.7)	6 (1.4)	11 (4.3)	8 (3.0)	11 (6.1)
Diarrhoea	63 (4.8)	60 (6.0)	33 (4.8)	24 (4.9)	14 (3.3)	4 (1.6)	6 (2.3)	7 (3.9)
Headache	64 (4.8)	55 (5.5)	32 (4.7)	32 (6.5)	18 (4.2)	16 (6.3)	10 (3.8)	6 (3.4)
Myalgia	54 (4.1)	57 (5.7)	33 (4.8)	13 (2.6)	20 (4.7)	9 (3.5)	9 (3.4)	9 (5.0)
Hypertension	47 (3.5)	39 (3.9)	27 (4.0)	19 (3.9)	25 (5.8)	13 (5.1)	16 (6.1)	7 (3.9)
Dizziness	43 (3.2)	38 (3.8)	31 (4.5)	18 (3.7)	22 (5.1)	11 (4.3)	14 (5.3)	10 (5.6)
Pain in extremity	43 (3.2)	30 (3.0)	23 (3.4)	25 (5.1)	12 (2.8)	4 (1.6)	5 (1.9)	5 (2.8)
Fall	35 (2.6)	30 (3.0)	21 (3.1)	25 (5.1)	10 (2.3)	8 (3.1)	6 (2.3)	2 (1.1)
Accidental overdose	16 (1.2)	14 (1.4)	10 (1.5)	7 (1.4)	30 (7.0)	23 (9.0)	9 (3.4)	13 (7.3)

SAE, serious adverse event; TEAE, treatment-emergent adverse event.
