# Supplementary material for: Efficacy of alirocumab according to background statin type and dose: pooled analysis of 8 ODYSSEY Phase 3 clinical trials
Source: Sci Rep. 2017 Apr 4;7:45788. doi: 10.1038/srep45788 (PMC5379546; doi:10.1038/srep45788)
Supplement: Supplementary Information [file srep45788-s1.pdf]

Supplementary Material

Efficacy of alirocumab according to background statin type and dose: pooled analysis of 8 ODYSSEY Phase 3 clinical trials

Alberico L. Catapano, L. Veronica Lee, Michael J. Louie, Desmond Thompson, Jean Bergeron, Michel Krempf

Supplementary Table 1. Further details of statin dose usage

Data are n (%)		Pool 1 HIGH FH + LONG TERM (n=2448)		Pool 2 COMBO I + FH I + FH II (n=1051)		Pool 3 COMBO II + OPTIONS I + OPTIONS II (n=1130)	
**Statin**	**Dose**	**ALI (n=1625)**	**PBO (n=823)**	**ALI (n=699)**	**PBO (n=352)**	**ALI (n=686)**	**EZE (n=444)**
**Moderate-dose statin**		542 (33.4)	271 (32.9)	108 (15.5)	43 (12.2)	208 (30.3)	152 (34.2)
Atorvastatin	20 mg	138 (8.5)	62 (7.5)	19 (2.7)	7 (2.0)	83 (12.1)	66 (14.9)
	30 mg	2 (0.1)	0	0	0	1 (0.1)	0
Rosuvastatin	10 mg	66 (4.1)	34 (4.1)	14 (2.0)	5 (1.4)	61 (8.9)	62 (14.0)
	15 mg	0	1 (0.1)	1 (0.1)	0	0	0
Simvastatin	40 mg	332 (20.4)	171 (20.8)	73 (10.4)	30 (8.5)	62 (9.0)	22 (5.0)
	50 mg	0	1 (0.1)	0	0	0	0
	60 mg	4 (0.2)	2 (0.2)	1 (0.1)	1 (0.3)	1 (0.1)	2 (0.5)
**Low-dose statin**		297 (18.3)	151 (18.3)	47 (6.7)	26 (7.4)	47 (6.9)	27 (6.1)
Atorvastatin	5 mg	0	0	2 (0.3)	0	0	0
	5.7 mg	1 (0.1)	0	0	0	0	0
	10 mg	67 (4.1)	33 (4.0)	7 (1.0)	3 (0.9)	5 (0.7)	6 (1.4)
Rosuvastatin	2.5 mg	1 (0.1)	0	0	2 (0.6)	0	0
	5 mg	22 (1.4)	10 (1.2)	11 (1.6)	6 (1.7)	9 (1.3)	1 (0.2)
Simvastatin	5 mg	2 (0.1)	1 (0.1)	0	1 (0.3)	0	0
	10 mg	45 (2.8)	21 (2.6)	3 (0.4)	3 (0.9)	5 (0.7)	4 (0.9)
	20 mg	153 (9.4)	85 (10.3)	24 (3.4)	11 (3.1)	28 (4.1)	16 (3.6)
	30 mg	6 (0.4)	1 (0.1)	0	0	0	0

ALI, alirocumab, EZE, ezetimibe, PBO, placebo.

Supplementary Table 2. Absolute change from baseline in LDL-C at Week 24: Subgroup analysis by statin type and dose

			Absolute change from baseline LS mean (SE)	
**Study pool**	**Subgroup**	**N**	**Control**	**Alirocumab**
A) Atorvastatin				
ALI 150 vs PBO	10 mg	98	–4.2 (6.1)	–69.6 (4.3)
	20 mg	195	–8.2 (4.6)	–70.9 (3.0)
	40 mg	362	–1.0 (3.3)	–73.7 (2.3)
	80 mg	264	–4.6 (4.0)	–76.2 (2.6)
ALI 75/150 vs PBO	40 mg	140	–1.7 (5.2)	–67.6 (4.1)
	80 mg	194	10.1 (4.6)	–65.4 (3.3)
ALI 75/150 vs EZE	20 mg	145	–23.8 (4.2)	–50.5 (3.7)
	40 mg	260	–25.6 (3.2)	–55.8 (2.6)
	80 mg	136	–21.3 (4.8)	–53.2 (3.4)
B) Rosuvastatin				
ALI 150 vs PBO	5 mg	30	6.6 (11.7)	–75.1 (8.8)
	10 mg	100	–14.2 (6.6)	–80.8 (4.7)
	20 mg	213	–9.3 (4.5)	–80.3 (3.2)
	40 mg	255	–2.6 (3.9)	–80.1 (3.0)
ALI 75/150 vs PBO	20 mg	149	–0.2 (5.9)	–67.3 (3.8)
	40 mg	302	11.1 (3.8)	–63.8 (2.7)
ALI 75/150 vs EZE	10 mg	121	–21.5 (4.9)	–56.3 (4.8)
	20 mg	221	–24.3 (3.9)	–52.3 (3.2)
	40 mg	49	–21.4 (8.9)	–53.9 (6.3)
C) Simvastatin				
ALI 150 vs PBO	10 mg	68	2.1 (7.0)	–66.2 (4.8)
	20 mg	233	–1.7 (3.6)	–73.0 (2.7)

	40 mg	495	–4.2 (2.5)	–74.6 (1.8)
	80 mg	76	–1.7 (5.8)	–71.9 (5.0)
ALI 75/150 vs PBO	20 mg	35	–12.9 (10.6)	–51.8 (7.2)
	40 mg	104	–5.3 (6.2)	–62.1 (4.1)
	80 mg	30	13.5 (9.1)	–54.1 (8.9)
ALI 75/150 vs EZE	20 mg	44	–20.1 (7.9)	–42.0 (6.1)
	40 mg	79	–22.7 (6.6)	–58.3 (4.1)
	80 mg	15	–19.8 (17.1)	–47.4 (10.1)

ALI, alirocumab; EZE, ezetimibe; LDL-C, low-density lipoprotein cholesterol; LS, least squares; PBO, placebo; SE, standard error.
